# Elliptical Supercritical Lens for Shaping Sub-Diffractive Transverse Optical Needle

**DOI:** 10.3390/nano13020242

**Published:** 2023-01-05

**Authors:** Jian Lei, Minghui Wang, Jin Wu, Hui Duan, Kun Zhang, Sicong Wang, Yaoyu Cao, Xiangping Li, Fei Qin

**Affiliations:** Guangdong Provincial Key Laboratory of Optical Fiber Sensing and Communications, Institute of Photonics Technology, Jinan University, Guangzhou 510632, China

**Keywords:** planar diffracted lens, elliptical supercritical lens, sub-diffractive limited

## Abstract

Supercritical lens can create a sub-diffraction-limited focal spot in the far field, providing a promising route for the realization of label-free super-resolution imaging through the point scanning mechanism. However, all of the reported supercritical lenses have circular shape configurations, and produce isotropic sub-diffraction-limited focal spots in the focal plane. Here, we propose and experientially demonstrate a sub-diffraction transverse optical needle by using an elliptical supercritical lens. Through breaking the circular symmetry and introducing ellipticity to the lens, a uniform sub-diffractive transverse optical needle with lateral length and width of 6λ/NA and 0.45λ/NA, respectively, was successfully created in the focal plane. Further, elliptical sector-shape cutting with an optimized apex angle of 60 degrees can lead to suppressed subsidiary focusing for improved uniformity and condensed field intensity of the transverse optical needle. The demonstration of sub-diffractive transverse optical needle with a high aspect ratio (length to width ratio) of 13:1 may find potential applications in line-scanning microscopy for video-rate label-free super-resolution imaging, and also enable advances in the fields from laser manufacturing to optical manipulation.

## 1. Introduction

Line scanning confocal microscopy is a cutting-edge technology for achieving high imaging throughput while retaining the essential advantage of conventional confocal microscopy [1,2,3]. By using a transverse optical needle to replace the isotropic focal spot, the image acquisition rates up to 100 fps have been successfully achieved by line-scanning confocal microscopy [4]. With such a system, the imaging process can significantly release the compromise between the imaging quality, field of view, and acquisition speed, then across the obstacle of in vivo imaging of living tissues. The line-scanning working manner can also be applied in the fields of light-sheet microscopy [5] and high-definition fluorescent micro-optical sectioning tomography, etc. [6]. However, the lateral size of the transverse optical needle created by the classical technique is bounded by the diffraction limit barrier; therefore, the imaging capability of the line-scanning confocal microscopy cannot meet the advanced requirement of sub-diffraction-limited property in biological imaging process. In addition, the transverse optical needle is usually created by utilizing the cylindrical lens and objective lens, making the line-scanning confocal system bulky and with a low level of integration. To eliminate these obstacles, one possible approach relies on the planar diffractive metalens, which could modulate the intensity distribution on the focal plane in light of specific requirements, and could also remarkably simplify the bulky optical system.

Sub-diffraction-limited planar metalens, represented by the superoscillatory lens (SOL) and supercritical lens (SCL), have attracted considerable attention for their extraordinary light modulation capability and have rapidly advanced in terms of performance and functionality [7,8,9,10,11,12,13]. Through delicately controlling the interference effect, a sub-diffraction-limited focal spot can be formed in the focal plane, then inspiring versatile applications in the aspect of super-resolution telescope, nanometrology, optical nanofabrications, etc. [14,15]. It is particularly important to emphasize that the sub-diffraction-limited focal spot could also be used for the label-free super-resolution imaging through combining it with the confocal microscopy configurations [16,17,18]. The superior imaging property and optical sectioning capability was realized through a point-by-point scanning across the specimen. However, all the reported SOL and SCL produced an isotropic sub-diffraction-limited focal spot in the focal plane, due to their circular symmetric configuration. Thus, the SOL- and SCL-based super-resolution imaging processes have the similar deficiency with conventional point-scanning confocal microscopy, as well as its derivative technologies, in terms of low imaging throughput [19,20,21,22]. Therefore, expanding the isotropic sub-diffraction limited focal spot to a sub-diffraction limited transverse optical needle may potentially bring about benefits for the realization of line-scanning super-resolution imaging with high throughput.

In this work, we proposed and experimentally demonstrated a new type of supercritical lens (SCL) with an elliptical configuration for shaping a transverse optical needle in the focal plane with lateral size in the sub-diffraction-limited domain. Such a lens with a binary amplitude-type elliptical configuration can be conveniently fabricated by the standard nanofabrication technique. Through controlling the ellipticity of the lens configuration (aspect ratio of long axis to short axis), the length of the transverse optical needle can be reasonably controlled. A transverse optical needle with a lateral length of 7λ and sub-diffractive width of 0.45λ/NA (NA is the numerical aperture) has been successfully demonstrated. Further, elliptical sector-shape cutting with an optimized apex angle of 60 degrees can suppress subsidiary focusing for improved uniformity and condensed field intensity of the transverse optical needle. Comparing it with the cylindrical planar metalens, our elliptical supercritical lens achieved eight times higher intensity in the needle region under the same optimization conditions. Such an elliptical supercritical lens may pave the path for the realization of line-scanning confocal microscopy with video-rate super-resolution imaging capability. Our demonstration may also attractively offer the prospect of advances in the fields, from laser manufacturing to optical manipulation.

## 2. Results

### 2.1. Optimization Algorithm for Modified Elliptical SCL

**Figure 1:**
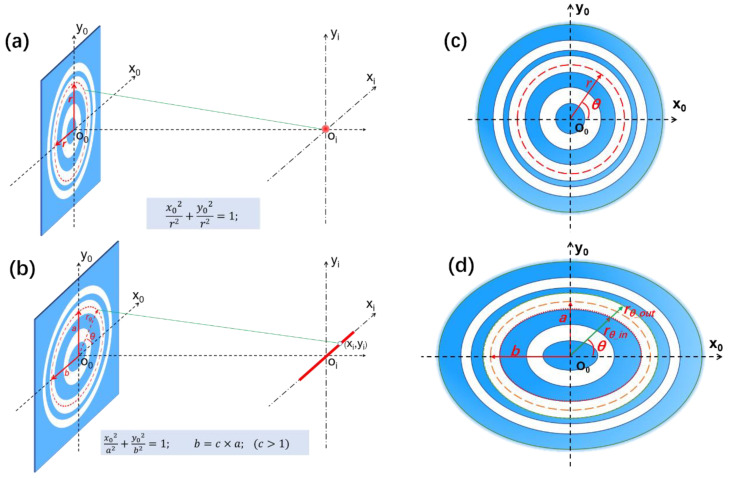
Schematic comparison between the circular and elliptical configuration of planar metalens. (**a**) shaping an isotropic focal spot by a circular planar metalens; (**b**) shaping a transverse optical needle in the focal plane by an elliptical planer metalens, where a and b are the semi-minor and semi-major axis length; (**c**) parameters of a planar metalens with circular configuration, where r is the radius of each transparent belt, and *θ* is the azimuthal angle; (**d**) parameters of a planar metalens with elliptical configuration, where the *r_θ_in_* and *r_θ_out_* indicate the inner and outer radial length of a transparent elliptical belt at the angle *θ*.

Ellipse is one of the most fundamental shapes with interesting properties in geometrical and physical fields. In the optical domain, rays of light originating at one of the foci of an elliptical mirror will be converged at the other focus after reflecting off the surface. Using this feature, the metal halide lamps used in microscopy are usually equipped with elliptical reflectors to generate a concentrated spot of light by embedding the bulb at one focus of the ellipse. However, almost all of the reported planar metalenses, including zone-plate-type [23,24,25,26], metasurface-type [9,27,28,29,30] and photosieves-type [31,32,33], have been of circular symmetric topology, and then lead to isotropic sub-diffraction-limited focal spots in the focal plane, as shown in Figure 1a. The elliptical configuration has seldom been investigated in the construction of a diffractive lens, due to the complicated field modulation property. A planar metalens in an ellipse configuration with non-circular symmetric topology should produce an anisotropic field distribution on the designed plane, as schematically expressed in Figure 1b.

For a conventional planar metalens with a circular configuration, the structures can be easily designed in a cylindrical coordinate system by adopting the Rayleigh–Sommerfeld integral method in conjunction with the particle swarm optimization process [11,13,34]. The field modulation property on the focal plane for each circular belt could be obtained through the angular integration over 2*π* space with a fixed radial length, as shown in Figure 1c. However, the topology of an ellipse shape and field intensity distribution of the transverse optical needle does not conform to the circular symmetry, and hence, the diffraction algorithm in a conventional cylindrical coordinate system needs to be modified for this situation. In the mathematical sense, every ellipse with foci on the *x*-axis can be obtained by stretching a circle horizontally, while leaving the vertical scale unchanged. Beginning with a circle center at the origin of coordinates, an ellipse will be created by changing every (*x*, *y*) on the circle to (*cx*, *y*) with a horizontal stretch factor of c (*c* > 1). Then, the obtained ellipse can be expressed as Equation (1), where a and b are the semi-major axis length and semi-minor axis length, *r* is the radius of the original circle, and the stretch factor c is the ellipticity of the obtained ellipse, which represents the ratio of semi-major axis length to semi-minor axis length as *c* = *a*/*b*.

(1) $$\frac{x^{2}}{a^{2}}+\frac{y^{2}}{b^{2}}=\frac{x^{2}}{{\left(cr\right)}^{2}}+\frac{y^{2}}{r^{2}}=1,$$

Transforming the standard formula of ellipse from the rectangular coordinate into the cylindrical coordinate, the radial length *r_θ_* from any point on the ellipse with angle *θ* can be derived as:

(2) $$r_{\theta }=a/\sqrt{{\left(c*sin\theta \right)}^{2}+{\left(cos\theta \right)}^{2}},$$

where *θ* indicates the counterclockwise angle with respect to the *x*-axis from the line connecting the point on the ellipse to the original point, and the *r_θ_* is the radial length at the angle *θ*, as shown in Figure 1d.

**Figure 2:**
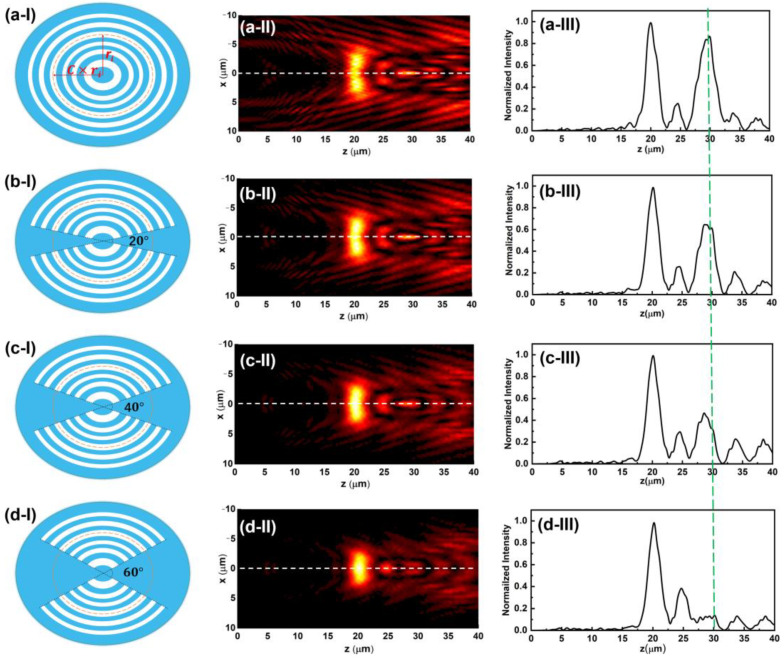
Comparison of light field distribution between elliptical SCL with different sector-shape cutting. (**a-I**,**b-I**,**c-I**,**d-I**) Scheme of the elliptical supercritical lens with cutting apex angle of 0 deg, 20 deg, 40 deg, and 60 deg, respectively; (**a-II**,**b-II**,**c-II**,**d-II**) the field distribution in the *x*-*z* plane with apex angle of the sector shape from 0 deg to 60 deg; (**a-III**,**b-III**,**c-III**,**d-III**) normalized intensity profiles along the optical axis for the corresponding elliptical SCL with different cutting sector. As the green dash line shows, the subsidiary focusing effect can be significantly suppressed while the apex angle of the sector-shape cutting region increases from 0 deg to 60 deg.

**Figure A1:**
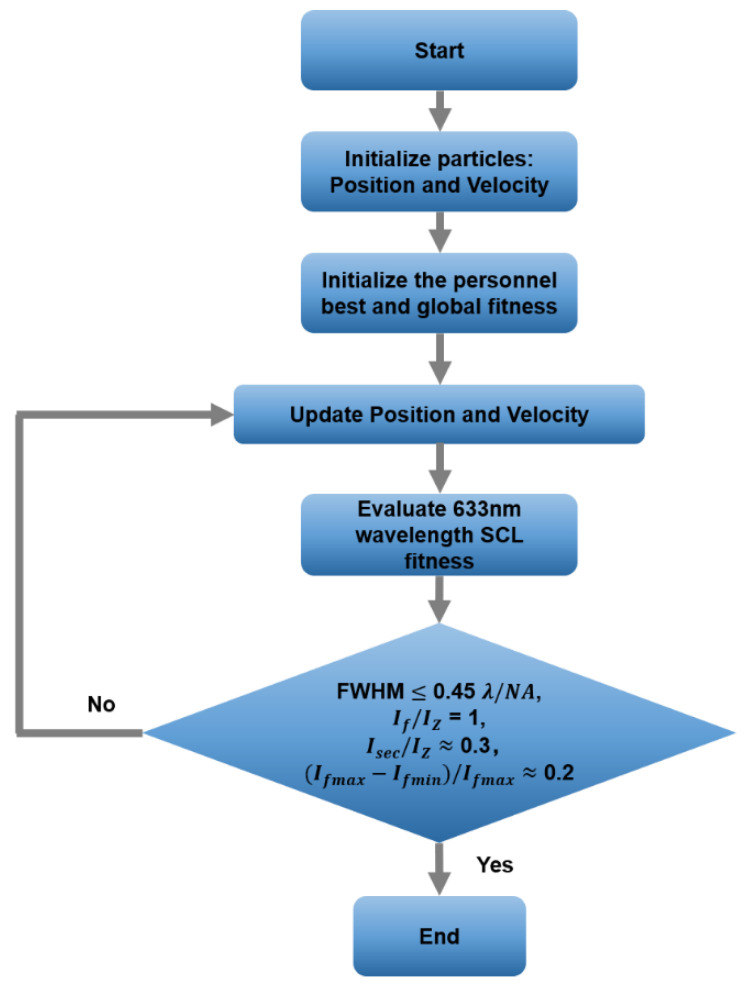
The optimization procedure for shaping sub-diffraction-limited transverse optical needle by an elliptical SCL.

**Figure A2:**
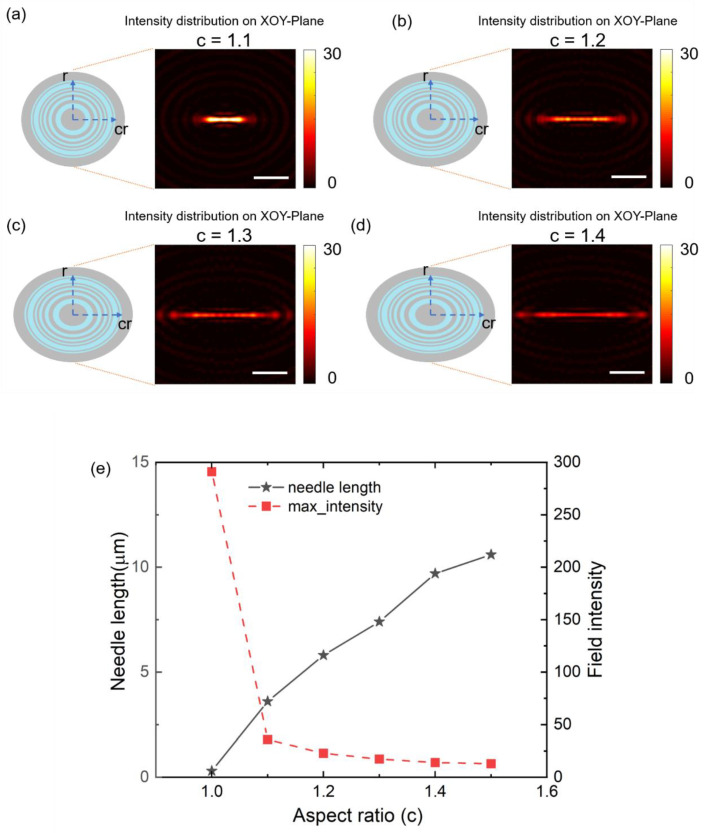
The scheme and intensity distribution of the transverse optical needle shaping by elliptical SCL with different aspect ratios (**a**–**d**). Scalebar: 350 nm. It can be seen from the statistic chart (**e**) that as the aspect ratio c increases from 1.0 to 1.4, the length of the transverse needle will be increased, but the light field intensity in the needle region will be dropped. To reach a compromise between the length and intensity of the optical needle, the aspect ratio value of 1.2 is chosen in our design.

Based on the above analysis, a modified Rayleigh–Sommerfeld (RS) diffraction integral method in a cylindrical coordinate applied to the ellipse pattern has been developed, as expressed in Equation (3). Comparing with the RS equation applied to the circular shape, the integral limits of the radius have been changed from a fixed value to an angle-dependent variable value according the elliptical equation for each transparent belt in cylindrical coordinates.

(3) $$U\left(\rho ,\theta ,z\right)=-\frac{1}{2\pi }\int_{r_{\theta \_in}}^{r_{\theta \_out}}\int_{0}^{2\pi }U_{0}\left(r_{\theta },\theta \right)\exp\left(iknR\right)*\frac{iknR-1}{R^{3}}*zr_{\theta }dr_{\theta }d\theta$$

where *U*_0_ is the incidence light field, *R* is the distance between two points in lens plane and focal plane, *r_θ_in_* and *r_θ_out_* indicate the inner and outer radial length of a transparent elliptical belt at the angle *θ*, respectively. Combined with the particle swarm optimization algorithm, a binary amplitude-type elliptical SCL could be successfully designed through tuning the position and width of each elliptical belt, as schematically shown in Figure 2a–I. The detailed analysis is shown in Appendix A.1 and Figure A1. Moreover, for an elliptical supercritical lens, the length of the created transverse optical needle heavily relies on the ellipticity of the structures, as the simulation results show in Figure A2 in Appendix A. To balance the length and intensity of the transverse optical needle, the ellipticity *c* = 1.2 is selected in the lens design. 

The simulated focusing properties of an elliptical SCL with a focal length of 20 μm for parameter analysis purposes is shown in Figure 2aI–aIII. The illuminating light we used in the simulation is a 633 nm linear polarized laser with polarization direction along the major axis of the ellipse. As the field intensity in the XOZ plane depicted in Figure 2aII shows, a transverse optical needle can be successfully created at the focal plane of *z* = 20 μm, as marked by the white dash line. However, a series of subsidiary focused light field appears around the designed transverse needle, which severely deteriorates the uniformity in the optical needle region and the purity along the optical axis, as shown in panel III. Such an effect can mainly be attributed to the elliptical geometric configuration. Ellipse shape has an angle-dependent variable radial length. On each transparent elliptical belt, the light field component with the same spatial frequency diffracted from the minor axis and major axis directions will be converged on different *z* positions. Thus, affected by this distinguished configuration, the intricate field intensity distribution will be created in the far field when an ellipse shape is used to construct a diffractive lens. To eliminate this phenomenon, a pair of sector-shape cutting regions are introduced to change the elliptical structure morphology. The modified elliptical supercritical lens is schematically shown in Figure 2bI–bIII,dI–dIII. As we can see from the simulation results, the subsidiary focusing effect can be significantly suppressed, while the apex angle of the sector-shape cutting region increases from 0 deg to 60 deg. Although the length of the transverse optical needle decreased, the light intensity in the needle region does not have an obvious change. It is noted that there are a series of moderate intensity peaks in the region of *z* = 25 μm to 40 μm. Nevertheless, the relative intensity of those peaks is below 0.2, which would not have a significant influence on the practical applications. To keep a proper balance between the needle length and subsidiary focusing effect, the elliptical sector-shape cutting with an apex angle of 60° in both sides along the major axis could produce the best performance. (For detail, refer to Appendix A.2). In addition, the modified elliptical SCL also shows additional benefits in the aspect of uniformity of the transverse optical needle.

### 2.2. Fabrication and Optical Characterization

**Figure 3:**
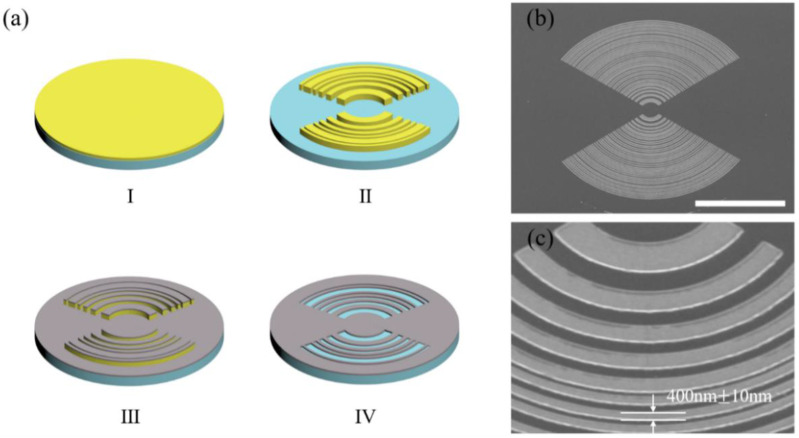
Schematic representation of the fabrication procedure. (**a**) The fabrication process of elliptical SCL: (I) spin coating with PMMA a4; (II) EBL patterning; (III) Ti Evaporation; (IV) lift off; (**b**) the SEM image of processing structure. Scale Bar: 50 μm; (**c**) the sectional zoom-in view of the elliptical SCL.

Under the guidance of the above theoretical analysis, a modified elliptical supercritical lens conforming to the simulation and experimental conditions was re-designed and fabricated, and its capability for shaping the transverse optical needle has been experimentally validated. The focal length *f* = 30 μm is chosen for the designed elliptical SCL at a wavelength of 633nm, and the numerical apertures are set at NA = 0.85. The entire lens consists of 40 transparent elliptical belts, and the radial lengths of the outmost semi-minor axis (*r*) and semi-major axis (*cr*) are 49.44 μm and 59.33 μm, respectively. The width of the transparent elliptical belts is variable, with the smallest value of 0.4 μm. The geometric parameters for the designed binary amplitude elliptical SCL is presented in Table A1 in Appendix A. The radial length r shown in Table A1 is the semi-minor axis, and the length of the semi-major axis for each belt can be written as 1.2*r*. Since the design pattern has a binary amplitude configuration with the smallest feature size of 400 nm, it can be easily fabricated by the standard lift-off process. The lens is patterned on the PMMA photoresist by electron beam lithography (EBL) firstly. Then, a layer of 100 nm titanium was deposited on the pattern by using an electron beam evaporator. After the lift-off process, the final supercritical lens with binary amplitude type and elliptical configuration was obtained. Figure 3a is the schematic representation of the fabrication procedure; for further details about the fabrication process refer to Appendix A.3. The scanning electron microscopy image of the fabricated elliptical SCL is shown in Figure 3b. The zoom-in view presented in Figure 3c indicates that the fabrication error can be controlled under +/−10nm to guarantee the focusing performance without deviation from the theoretical results.

**Figure 4:**
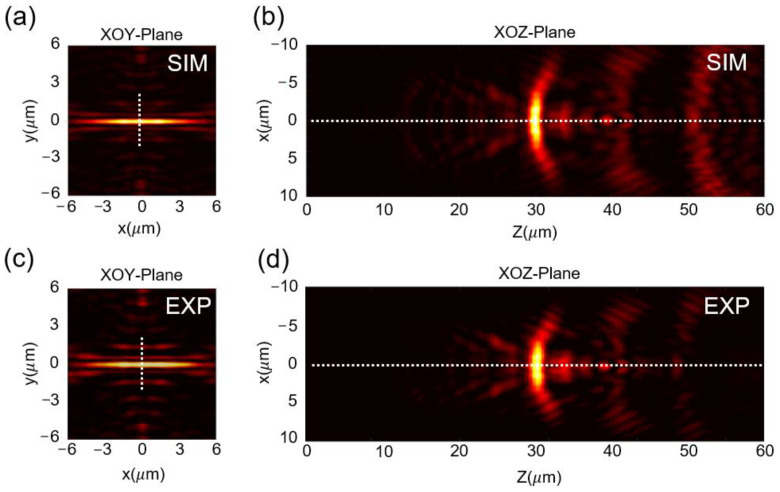

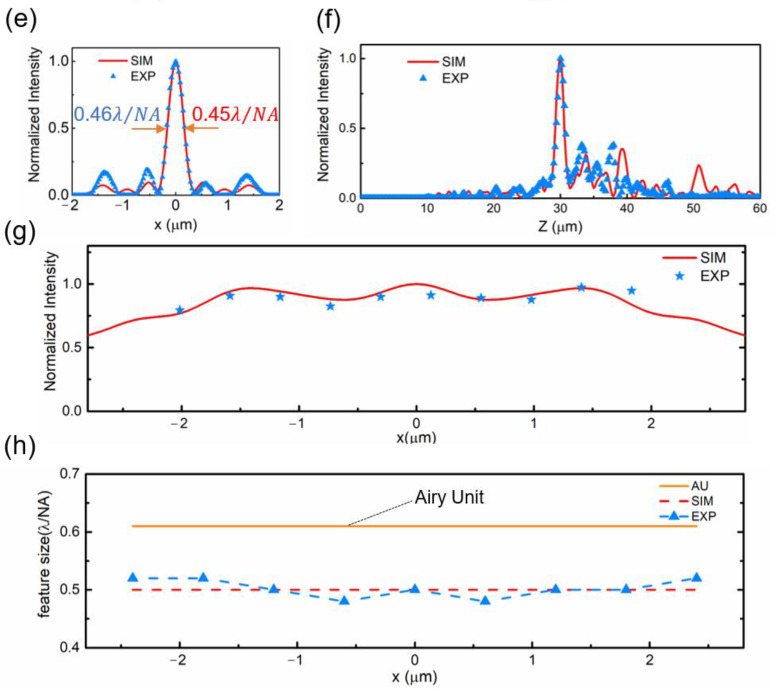
Light field distribution of the modified elliptical SCL. (**a**,**b**) Simulated result of the transverse optical needle in the XOY plane (**a**) and the XOZ plane (**b**) shaped by the modified elliptical SCL; (**c**,**d**) experimental result of the transverse optical needle in the XOY plane (**c**) and the XOZ plane (**d**); (**e**) intensity profiles of the transverse optical needle in the perpendicular direction, which is marked by the white dashed line in Figure 4a,c; (**f**) axial intensity distribution from the simulation (red line) and the experiment (blue dash symbol), which is marked by the white dash lines in Figure 4b,d; (**g**) intensity profiles along the transverse optical needle from the simulation (red line) and experimental results (blue star symbol); (**h**) the lateral sizes in a horizontal direction, which show a uniform sub-diffraction-limited optical needle.

Optical characterization was carried out by a customized microscope imaging system, as schematically shown in Figure A4 in Appendix A.4. A linear polarized He-Ne laser with 633 nm wavelength was applied to illuminate the fabricated elliptical SCL from the substrate side, then collect the diffracting pattern by a high quantum efficiency CMOS camera. The polarization state of the laser beam is set along the *x*-axis and in line with the major axis of the ellipse. The convergent sub-diffraction-limited transverse optical needle was formed in the focal plane along the major axis direction. The simulated and measured intensity distributions at the focal plane of *z* = 30 μm away from the SCL are depicted in Figure 4a–d, which clearly shows that a 4 μm-length (~6λ/NA) transverse optical needle in the focal plane has been obtained. Notably, the length of the optical needle depends on the size of the geometric dimension of the lens. Even a longer optical needle is possible in case a larger-size elliptical supercritical lens and high ellipticity value are selected. The line intensity profile in a perpendicular direction to the optical needle is plotted in Figure 4e. The lateral size of the transverse optical needle is 0.45λ/NA and 0.46λ/NA in simulation and experiment results, respectively, which exhibits a sub-diffraction-limited property. The field distribution in the XZ plane of the simulation and experimental results are presented in Figure 4b,d. The intensity profile along the white dashed line clearly depicted that the transverse optical needle dominates the entire diffraction region, as shown in Figure 4f.

Moreover, the uniformity, in the aspects of intensity distribution and lateral size along the needle region, is another important feature for the transverse optical needle. Apparently, as we can see from Figure 4g, the transverse optical needle could basically keep the field intensity in a constant, ranging from −2.0 to 2.0 μm along the needle region. The FWHM values in different positions along the optical needle are presented in Figure 4h, which clearly shows the uniform sub-diffraction-limited property within the needle region. The experimental measured lateral size of the transverse optical needle varies from 0.44λ/NA to 0.46λ/NA within the entire optical needle, showing great potential for the line-scanning super-resolution imaging application.

## 3. Discussion

**Figure 5:**
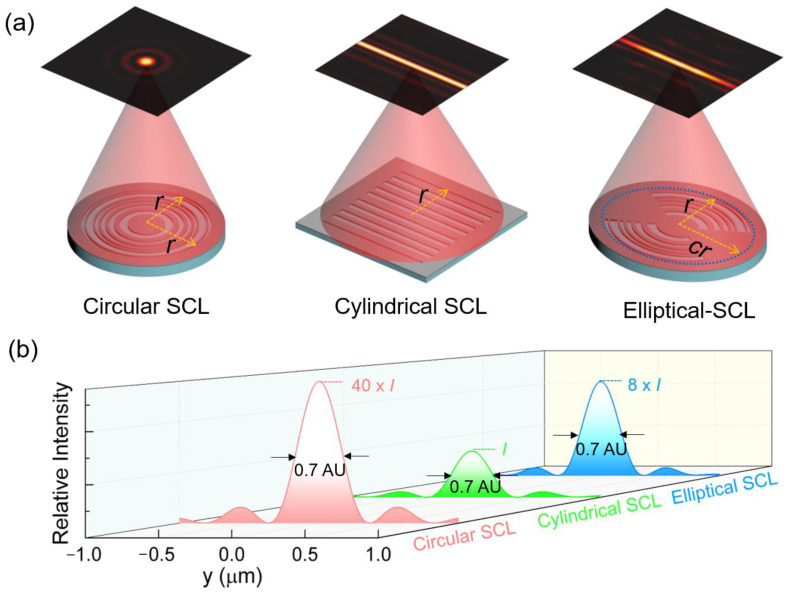
Comparison of three different types of supercritical lens. (**a**) Schematic diagram of sub-diffraction-limited focusing of different planar diffractive lenses, including a conventional circular supercritical lens, a 1D grating-type cylindrical supercritical lens, and an elliptical supercritical lens; (**b**) the relative intensity of the focal spot created by the circular SCL and transverse needles created by the cylindrical SCL and elliptical SCL. AU stand for Airy units; that is the radius of the Airy spot in the diffraction limited optical system. The transverse optical needle created by the elliptical SCL is shorter than the cylindrical SCL, but with eight times higher field intensity in the needle region.

**Figure A3:**
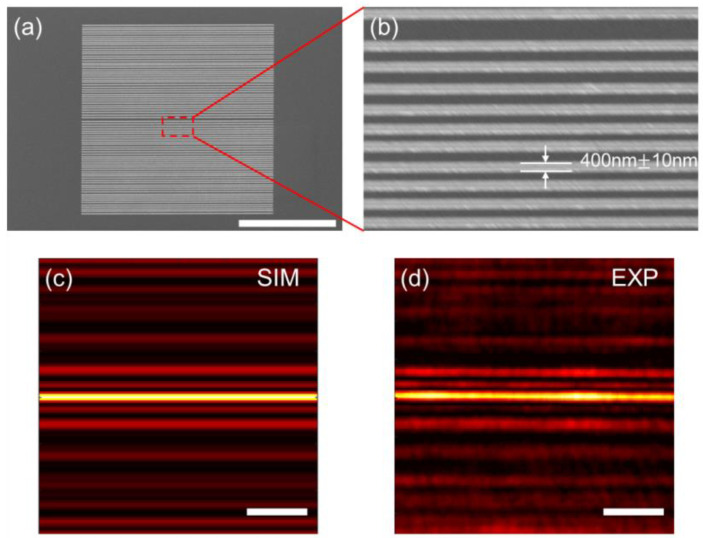
(**a**,**b**) The SEM image and its sectional zoom-in view of the grating-type cylindrical supercritical lens, scalebar: 50 μm; (**c**,**d**) the field intensity distribution in the XOY plane of the transverse optical needle created by the grating-type cylindrical SCL for the simulated result (**c**) and the experimental result (**d**), scalebar: 3 μm.

**Figure A4:**
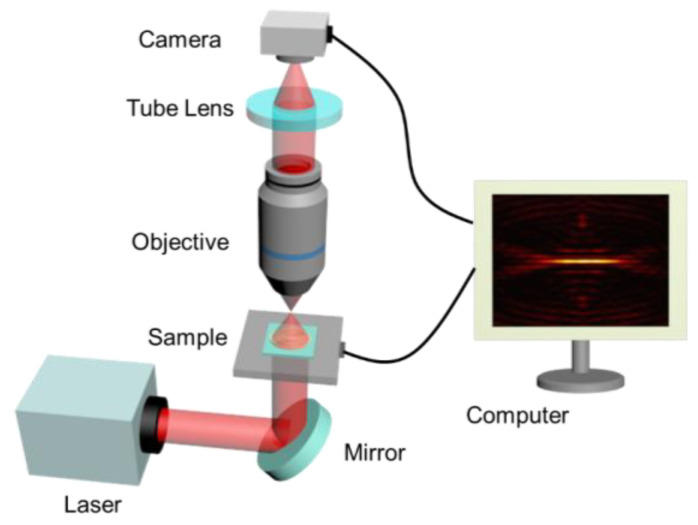
Schematic illustration of the optical characterization system.

Besides the circular and elliptical shape, planar metalens could also be constructed in a cylindrical configuration, and a different configuration will give different light field modulation capabilities. It has been demonstrated that planar metalens with a cylindrical configuration could also create a uniform transverse optical needle in the focal plane with subwavelength lateral size [35]. Compared with such type configurations, the transverse optical needle created by our modified elliptical supercritical lens has much higher field intensity in the needle region. To validate this argument, a comparison experiment was performed between our modified elliptical supercritical lens and a grating-type cylindrical supercritical lens under the same optimization conditions. A binary amplitude cylindrical supercritical lens with a scale of 100 μm × 100 μm was designed and experimentally fabricated. The SEM imaging and its sectional view of the fabricated cylindrical supercritical lens are shown in Figure A3a,b in Appendix A. As the simulation and experimentally measured results show in Figure A3c,d in Appendix A, a transverse optical needle can really be obtained in the focal plane when the 633 nm laser beam impinges on the pattern from a He-Ne laser. The lateral size of the transverse needle conforms to the design value, and the length of the needle region is essentially the same as the scale of the pattern along the horizontal direction, since the diffractive wave has no constriction along the horizontal direction. By contrast, although our modified elliptical supercritical lens cannot match the cylindrical counterpart in the aspect of the length of the transverse optical needle, our results have significant advantage in terms of the field intensity in the needle region. As schematically presented in Figure 5, the modified elliptical supercritical lens could bundle more light energy into the needle region, and boost the needle intensity much higher. The field intensity in the needle region of elliptical supercritical lens is 8 times and 7.5 times higher than the cylindrical supercritical lens in the simulation and experimental results, respectively, which makes it more feasible in practical applications.

## 4. Conclusions

In summary, we proposed an elliptical supercritical lens which could generate a sub-diffraction-limited transverse optical needle in the focal plane. Contrary to the previously demonstrated planar metalens with circular symmetry, the demonstrated elliptical supercritical lens consists of a series of concentric ellipse configurations. Such a type of planar metalens was designed by a modified Rayleigh–Sommerfeld diffraction integral algorithm in conjunction with the particle swarm optimization technique. The experimental demonstrations have verified that a 7λ-long transverse optical needle with lateral size of 0.46 λ /NA has been obtained in the focal plane 30 μm away from the lens plane. The light field distribution of a uniform intensity in the transverse optical needle region has shown significant advantage in the aspect of field intensity compared with the cylindrical sub-diffraction-limited planar metalens under the same conditions. The abovementioned unique property means our elliptical SCL has important value in the application of line-scanning super-resolution confocal microscopy. In addition, it can also serve as an ideal way in future applications, such as in ultra-precision optical micro-manipulation, optical data storage, etc.

## Data Availability

The data presented in this study are available on request from the corresponding author.

## Appendix A

### Appendix A.1. Design of Binary Amplitude Elliptical Super-Critical Lens

By using the modified RS diffraction integral method, a binary amplitude-type elliptical supercritical lens was designed. The optimization algorithm we used is the particle swarm optimization algorithm (PSO). The length of the semi-minor axis of the outermost elliptical belt is ~50 μm, and the numerical aperture is set at NA = 0.85. The minimum belt width is set at 400 nm to facilitate the nanofabrication process. Regarding the transverse optical needle preparing for the application in line-scanning super-resolution imaging, there are three important characteristics that need to be met simultaneously: sub-diffraction-limited lateral size, uniform field distribution, and lower subsidiary focusing effect around the needle region. All those features need to be considered during the optimization procedure. The optimization procedure for designing elliptical SCL is shown in Figure A1.

It is noted that the ellipticity factor is the essential parameter distinguishing an ellipse from a circle. The length of the created transverse optical needle by the elliptical supercritical lens heavily relies on the ellipticity of the structures. As the simulation results show in Figure A2, the property of the transverse optical needle will be changed along with the ellipticity factor alteration. Larger ellipticity will give a longer transverse optical needle, but lower intensity in the needle region. A proper elliptical factor should be selected in the lens design.

The optimization process can be divided into two steps as follows: (I) generate the position and the width data of a set of belt positions randomly by using the particle swarm optimization algorithm, and then use a set of parameters to form the corresponding binary amplitude elliptical supercritical lens at a wavelength of 633 nm for evaluating the focused performance. Different from the supercritical lens in the circular configuration, the elliptical structure can make the focused light field spread along x axis, so we calculate the integral of the light field intensity (I_Z_) of each point on the optical axis within a certain length along the *x*-axis; (II) compare the value obtained from the evaluation function with the final value. If the precision of the target value is reached, the optimization is completed, and the optimized parameters are output. Otherwise, it will enter the next generation until the precision of the target value is reached. The optimized parameters of the elliptical supercritical lens are shown in Table A1.

**Table A1:** The position information of each belt for elliptical supercritical lens.

No	Opaque		Transparent		No	Opaque		Transparent	
	0		1			0		1	
	Rin/μm	Rout/μm	Rin/μm	Rout/μm		Rin/μm	Rout/μm	Rin/μm	Rout/μm
1	0.00	4.37	4.37	6.20	21	29.38	30.17	30.17	30.57
2	6.20	7.61	7.61	8.81	22	30.57	31.28	31.28	31.68
3	8.81	9.87	9.87	10.84	23	31.68	32.43	32.43	32.83
4	10.84	11.74	11.74	12.58	24	32.83	33.33	33.33	33.73
5	12.58	13.38	13.38	14.14	25	33.73	34.20	34.20	34.60
6	14.14	14.57	14.57	14.97	26	34.60	35.09	35.09	35.49
7	14.97	15.58	15.58	15.98	27	35.49	35.99	35.99	36.39
8	15.98	16.46	16.46	16.86	28	36.39	37.00	37.00	37.40
9	16.86	17.72	17.72	18.12	29	37.40	38.14	38.14	38.54
10	18.12	18.76	18.76	19.16	30	38.54	39.04	39.04	39.44
11	19.16	20.00	20.00	20.40	31	39.44	40.01	40.01	40.41
12	20.40	20.93	20.93	21.33	32	40.41	40.93	40.93	41.33
13	21.33	22.16	22.16	22.56	33	41.33	42.22	42.22	42.62
14	22.56	23.16	23.16	23.56	34	42.62	43.07	43.07	43.47
15	23.56	24.34	24.34	24.74	35	43.47	44.16	44.16	44.56
16	24.74	25.14	25.14	25.54	36	44.56	45.08	45.08	45.48
17	25.54	26.13	26.13	26.53	37	45.48	46.11	46.11	46.51
18	26.53	27.17	27.17	27.57	38	46.51	47.00	47.00	47.40
19	27.57	28.07	28.07	28.47	39	47.40	47.99	47.99	48.39
20	28.47	28.98	28.98	29.38	40	48.39	49.04	49.04	49.44

### Appendix A.2. Introduction of the Sector-Shape Cutting Region

From the geometric perspective, the major difference between the circle and ellipse is that the ellipse shape has angle-dependent variable radial length. The same spatial frequency light diffracted from the minor axis direction and major axis direction will converged on different *z* positions. Thus, affected by this distinguished configuration of the elliptical belts, intricate field intensity distribution will be created in the far field when an ellipse shape is used to construct a diffractive lens. Besides the transverse optical needle in the designed focal plane, a series of subsidiary intensity peaks appeared in the focal region, as shown in Figure 2aI–aIII. To allow this phenomenon to obtain a uniform transverse optical needle, the elliptical structure morphology needs to be modified. As schematically shown in Figure 2bI–bIII,dI–dIII, a pair of sector-shape cutting regions were introduced. In the theoretical attempt, a sector shape with a different angle from 0 deg to 60 deg was simulated. It was found that the sector shape with 60 deg will give a better performance, where the subsidiary focused light field generated by the original ellipse structure will be effectively suppressed. In addition, the modified elliptical supercritical lens also shows additional benefits in the aspect of uniformity of the transverse optical needle. The diffraction field of the modified elliptical supercritical lens can be calculated with the Equation (A1) as follow:

(A1) $$U\left(\rho ,\theta ,z\right)=-\frac{1}{\pi }\int_{r_{\theta \_in}}^{r_{\theta \_out}}\int_{\frac{\pi }{6}}^{\frac{5\pi }{6}}U_{0}\left(r_{\theta },\theta \right)\exp\left(iknR\right)\times \frac{iknR-1}{R^{3}}\times zr_{\theta }dr_{\theta }d\theta ,$$

### Appendix A.3. Nanofabrication Process of the Binary Amplitude Supercritical Lens

The elliptical supercritical lens with binary amplitude configuration was fabricated by using electron-beam lithography, followed by a standard e-beam deposition and lift-off process, as shown in Figure 3. The structure was patterned on a 1mm-thick ITO substrate. After thoroughly cleaning the substrate with acetone, isopropyl alcohol (IPA), and deionized (DI) water, the positive electron beam resist PMMA A4 (MicroChem, Cook County, GA, USA) was spin coated at 2000 rpm on the prepared substrate with a thickness of 300 nm, and then baked on a hot plate for 3 min at 180 °C. The EBL system (EBPG5150, Raith, Best, The Netherlands) had an acceleration voltage of 100 keV and a dose of 510 μC/cm^2^. Since the smallest feature size of our pattern is 400 nm, proximity effect correction is unnecessary in the lithography process. After the developing process, a 100 nm-thick titanium (Ti) film was deposited on the sample by electron-beam evaporator (Explorer Coating System, Denton Vacuum, Moorestown, NJ, USA) with a deposition velocity of 0.1 nm/s. Subsequently, the sample was soaked in the stripping solution overnight for lift-off process, then the final sample was obtained. The fabricated elliptical supercritical lens was imaged by using a scanning electron microscope (Apreo HiVac, FEI, Hillsboro, OR, USA) with an accelerating voltage of 5 kV. As a control sample, a cylindrical supercritical lens with a similar size has been fabricated with the same procedure, as shown in Figure A3.

### Appendix A.4. Optical Characterization of the Patterned Supercritical Lenses

The optical characterization of the elliptical supercritical lens was performed by a self-built microscope, as schematically shown in Figure A4. A low power He-Ne linear polarized laser (DH-HN250P, 2 mW, Daheng Optics, Beijing, China) was applied as the illumination source. The wavelength is 633 nm, and the polarization state is in the x direction along the major axis of the ellipse. In experiments, the laser beam was illuminated on the binary amplitude type elliptical supercritical lens from the substrate side. The alignment between the laser beam and the SCL was performed by a motorized translation stage (PI, M687PILine, Karlsruhe, Germany). An objective lens with NA = 0.9 (Nikon, TU plan EPI 100X, Tokyo, Japan) was used to collect the transmitted light. The image of the transmitted light field was acquired and recorded by a high quantum efficiency CMOS camera (Nikon Qi2, Tokyo, Japan) in order to obtain the field intensity in the xy plane. A 2.5X extender tube is inserted in between the objective lens and CMOS camera to further increase the magnification. The intensity distribution in the XOZ plane was obtained by scanning the SCL along the *z* direction by a Piezo stage (PI, P-736.ZRN, Karlsruhe, Germany) with a stepwise of 200 nm, and then mapping the intensity distribution in the longitudinal plane.
